# Fatal Fulminant Hepatitis E in a Diabetic Patient on Metformin

**DOI:** 10.3390/diagnostics12102385

**Published:** 2022-09-30

**Authors:** Erika Peroni, Pierre Mora, Anne Motte, René Gerolami, Sarah Aherfi, Philippe Colson

**Affiliations:** 1 IHU Méditerranée Infection, 13005 Marseille, France; 2 Assistance Publique-Hôpitaux de Marseille (AP-HM), Hôpital Nord, Service de Réanimation, 13005 Marseille, France; 3 Assistance Publique-Hôpitaux de Marseille (AP-HM), Hôpital Timone, Service D’hépato-Gastro-Entérologie, 13005 Marseille, France; 4 Institut de Recherche pour le Développement (IRD), Microbes Evolution Phylogeny and Infections (MEPHI), Aix-Marseille Université, 13005 Marseille, France; 5 Assistance Publique-Hôpitaux de Marseille (AP-HM), 13005 Marseille, France

**Keywords:** hepatitis E, fulminant hepatitis, metformin, diabetes, death

## Abstract

Hepatitis E is mostly autochthonous in Western developed countries, eating pig-derived products being the most frequently documented source. Hepatitis E virus (HEV) infection is usually asymptomatic or self-limiting, but it can cause acute liver failure. HEV serological testing was performed using EUROIMMUN immunoenzymatic assays. HEV RNA in the serum was determined using an in-house real-time reverse transcriptase PCR procedure. The HEV genotype was determined through phylogenetic analysis after Sanger sequencing was performed using an in-house procedure. The case patient, an immunocompetent patient in his 60s with type 2 diabetes and no documented chronic liver disease, was hospitalized in February 2021 in an intensive care unit due to an initially unexplained coma. He presented metformin overdose and fulminant hepatitis E (HEV RNA in the serum was 4,140,000 copies/mL) that evolved toward death. The HEV genotype was 3f. We identified eight previous hepatitis E in diabetic patients, but with no metformin excessive plasma concentration, in the literature. Three patients were liver transplant recipients and three died. HEV infection can be severe and life-threatening in diabetic patients, which warrants HEV testing in this special population in the case of an altered general condition and/or liver cytolysis.

## 1. Introduction

For 15 years, Hepatitis E has been known to be mainly autochthonous in Western developed countries with a porcine reservoir and eating pig-derived products being the most frequently documented source [1]. The causative agent, hepatitis E virus (HEV), is a quasi-enveloped RNA virus classified as the species *Paslahepevirus balayani* in the genus *Paslahepevirus* (https://ictv.global/report/chapter/hepeviridae/hepeviridae/orthohepevirinae/paslahepevirus, accessed on 17 September 2022) [2]. The genotypes mainly found in Europe and other developed countries are genotypes 3 and 4 [1]. Although most often asymptomatic or self-limiting, hepatitis E causes acute liver failure in 0.5–4% of individuals and can be life threatening [1]. In addition, it can cause chronic hepatitis in immunocompromised patients and extra-hepatic manifestations, including neurological symptoms. Severe and fatal acute liver failure mainly occur in patients with underlying liver disease, but acute fulminant hepatitis was also reported [3]. HEV-associated mortality in developed countries is probably still underestimated. In France, it was reported that the numbers of cases, hospitalizations, and deaths during 2008–2013 were 68,007, 546, and 20 per year, respectively [4]. We previously described six fatal cases of hepatitis E between 2006–2015 in Marseille [3]. Here, we report HEV infection in a 69-year-old diabetic patient on metformin, leading to fatal acute fulminant hepatitis.

## 2. Patient and Methods

The case patient was hospitalized in a public university hospital of Marseille, southeastern France. HEV IgM and IgG antibodies were detected by an immuno enzyme assay (Euroimmun, Bussy Saint-Martin, France). HEV RNA in the serum was determined using an in house real-time reverse transcriptase PCR procedure [5]. Anti-hepatitis A virus (HAV) IgM and IgG were determined with the Architect assays (Abbott Laboratories, IL, USA). Anti-HAV IgM was also determined with the Vidas assay (bioMérieux, Meylan, France). HAV RNA in the serum was determined using an in house real-time reverse transcriptase PCR procedure [6]. The HEV genotype was determined through phylogenetic analysis and through the Hepatitis E Virus Genotyping Tool v0.1 (https://www.rivm.nl/mpf/typingtool/hev/, accessed on 17 September 2022) after Sanger sequencing was performed using an in-house procedure [5]. For phylogenetic analysis, the 10 sequences with the highest BLAST scores recovered from the NBCI GenBank nucleotide sequence databases (http://www.ncbi.nlm.nih.gov/nucleotide/, accessed on 17 September 2022) were incorporated in the phylogeny reconstruction, in addition to reference sequences for HEV genotypes [2] and the HEV sequence obtained from the present case. Nucleotide alignments were performed using the MUSCLE software (http://www.ebi.ac.uk/Tools/msa/muscle/, accessed on 17 September 2022). The evolutionary history was inferred in MEGA software (http://www.megasoftware.net/, accessed on 17 September 2022) using the Neighbor-Joining method and the Kimura 2-parameter method. The bootstrap test included 1000 replicates. All data were generated as part of the routine work at Assistance Publique-Hôpitaux de Marseille (Marseille university hospitals). This study was approved by the IHU Méditerranée Infection’s ethics committee (No. 2019-001).

## 3. Results: Case Report

**Figure 1:**
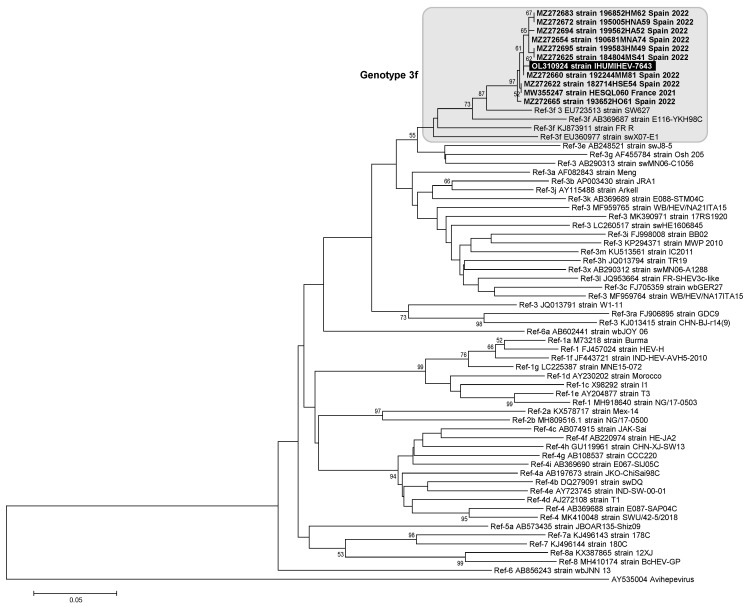
The fragment of the ORF2 gene encoding for the capsid protein was 315-nucleotide long and corresponded to nucleotides 6016–6330 of the HEV genome GenBank accession no. JQ953664. The HEV sequence obtained by Sanger sequencing from the present case is indicated by a white bold font and a black background. The 10 sequences with the highest BLAST scores recovered from the NBCI GenBank nucleotide sequence databases (http://www.ncbi.nlm.nih.gov/nucleotide/, accessed on 17 September 2022), indicated by bold font, were incorporated in the phylogeny reconstruction, in addition to reference sequences for the HEV genotypes [2]. Nucleotide alignments were performed using the MUSCLE software (http://www.ebi.ac.uk/Tools/msa/muscle/, accessed on 17 September 2022). The evolutionary history was inferred in the MEGA6 software (http://www.megasoftware.net/, accessed on 17 September 2022) using the Neighbor-Joining method and the Kimura 2-parameter method. The percentage of replicate trees in which the associated taxa clustered together in the bootstrap test (1000 replicates) is shown next to the branches. The tree is drawn to scale, with branch lengths in the same units as those of the evolutionary distances used to infer the phylogenetic tree; the scale bars indicate the number of nucleotide substitutions per site. Bootstrap values > 50% are labeled on the tree.

The patient was a 69-year-old man with type 2 diabetes treated with metformin (1000 mg thrice-a-day) and with arterial hypertension and dyslipidemia. He was hospitalized in February 2021 in an intensive care unit (ICU) for an initially unexplained coma. He was found unconscious at home with deterioration of general condition for 10 days, with fever, nausea, and vomiting, and 6 kg weight loss. He had no known liver disease, was not cirrhotic, did not report an excessive alcohol intake, and had a mean globular volume of red blood cells within the normal range. He exhibited no sign of chronic hepatitis, including no portal hypertension sign (neither splenomegaly, periumbilical collateral venous circulation, ascites, stellar angioma, nor hepatosplenomegaly). The thoraco–abdomino–pelvic scan showed no liver anomaly. At the time of hospitalization, the patient presented multiple organ failure with acute liver and renal failure, and neurological distress with consciousness loss (Glasgow score of 8). Biological examinations notably showed lactic acidosis and liver cytolysis with an alanine aminotransferase level (ALT) at 3079 IU/L (upper usual value, 50 IU/L), aspartate aminotransferase level (AST) at 1926 IU/L (upper usual value, 50 IU/L), gammaglutamyl transferase level at 308 IU/L, bilirubinemia at 51 µmol/L, and a prothrombin index at 24%. HEV diagnosis was based on positivity of anti-HEV IgM (serum-to-threshold optical density ratio (ODR) > 6) and IgG (5.57 IU/mL) and HEV RNA in serum (4,140,000 copies/mL). The other major causes of hepatitis, including hepatitis B, C, and D viruses, cytomegalovirus, Epstein–Barr virus, and herpes simplex virus were excluded by serology and/or PCR. Anti-hepatitis A virus (HAV) IgM (Architect assay: index, 5.46 (positivity threshold, 1.2) (Abbott Laboratories, IL, USA); Vidas assay: index, 0.91 (positivity threshold, 0.5) (bioMérieux, Meylan, France) and IgG (Architect assay: ODR = 7.9) were detected, but HAV RNA testing (in house real-time reverse transcriptase PCR [6]) was negative. This suggested either recent resolved HAV infection or false-positivity of anti-HAV IgM; no information was obtained regarding prior anti-HAV vaccine immunization. SARS-CoV-2 PCR was negative at symptom onset and upon admission to the ICU. Metformin plasma concentration was 33 µg/mL, whereas normal values are 1–4 µg/mL, and the concentration is considered toxic when ≥10 µg/mL. This caused acute renal failure, and led to hemodialysis that successfully fought biguanide intoxication. Liver transplantation was not performed due to major hemodynamic instability, despite the administration of norepinephrine, terlipressin, and epinephrine. Two days post-admission, ALT was 4549 IU/L, bilirubinemia was 70 µmol/L, prothrombin index was 11%, and the patient died. The HEV genotype was 3f (GenBank Accession no. OL310924) (Figure 1).

The patient had not been questionable upon admission and he lived alone. Whether he had consumed any pig-derived food product, including pig liver sausage, could not be determined. There was no documentation of recent travel or contact with pets. The family only described antecedents of chronic diarrhea.

## 4. Discussion

A few cases of hepatitis E were reported in patients with diabetes, which was considered a risk factor for severe progression due to HEV-related liver disease. We identified eight hepatitis E in diabetic patients in the literature, with no reported metformin overdosage or excessive plasma concentration. Two patients were liver-transplanted and two died. Ishiwata et al. reported the case of a 70-year-old diabetic HEV genotype-3-infected woman with asthenia and liver cytolysis (ALT, 2565 IU/L) who experienced full hepatitis E resolution [7]. The patient was receiving metformin, but without excessive dosage history. Wenter et al. reported the case of a 78-year-old immunocompetent diabetes patient, who presented with symptomatic infection by a genotype 3 virus and progressed to acute liver insufficiency [8]. Ribavirin treatment rapidly lowered the HEV RNA load and led to complete resolution of acute fulminant hepatitis E. Gynthersen et al. reported the case of a 50-year-old type 2 diabetic, alcoholic patient admitted for jaundice, fever, and a painful abdomen and who developed meningoencephalitis symptoms [9]. This patient gradually improved clinically within 8 days post-hospitalization. Inagaki et al. reported three cases of diabetic patients who were HEV-infected [10]. They included a 63-year-old man with spontaneously resolving acute hepatitis, a 58-year-old man with hepatic encephalopathy and acute fulminant hepatitis who survived, and a 62-year-old man with pre-existing liver cirrhosis aggravated by HEV who died of hepatic encephalopathy. Aherfi et al. reported the case of a 58-year-old type 2 diabetic patient with acute hepatitis caused by genotype 3f HEV who exhibited hepatic encephalopathy and died 5 days post-liver transplantation [3]. Finally, Fantilli et al. reported the case of a 56-year-old hypertensive type 2 diabetic patient with alcohol-related liver cirrhosis admitted for asthenia, jaundice, and acholia and who developed meningoencephalitis symptoms [11]. The patient was receiving metformin, but without reported excessive dosage. This patient evolved towards grade I hepatic encephalopathy and was transplanted three months post-HEV diagnosis, and then was transplanted and died one year later.

In the present case, the reasons for the fatal outcome associated with the liver injury are unclear. HEV infection is potentially life-threatening, particularly in patients with underlying liver disease [1]. We cannot in absolute exclude an acute-on-chronic liver failure, but we found no evidence in favor of prior chronic liver disease. The combination of HEV infection and metformin intoxication could explain the liver failure and fatal outcome. Metformin is not considered intrinsically hepatotoxic as it is not metabolized in the liver [12]. Nonetheless, metformin-associated hepatotoxicity has been reported in rare cases [12,13,14,15,16], with hepatocellular or cholestatic mechanisms being deemed to be the cause of liver injury [16]. A concomitant intake of other potentially hepatotoxic drugs was reported in some of these cases [12]. Here, the metformin plasma concentration was excessive, which might have aggravated liver injury, and reached toxic levels that can cause lactic acidosis. It is also worthy to note that anti-HAV IgM were detected in presence of IgG, but in the absence of HAV RNA, suggesting recent resolved HAV infection or a false-positivity of anti-HAV IgM. The co-existence of anti-HEV and anti-HAV IgM was reported in previous studies and was interpreted as HEV and HAV coinfections, but in the absence of HEV RNA and HAV RNA testing, in all but one study [17]. Here, whether recent HAV infection had occurred and impacted the clinical outcome at the time of hepatitis E associated with excessive metformin dosage remain unresolved questions.

## 5. Conclusions

Overall, the present case and the few previous reports suggest that HEV infection can be particularly severe and even life-threatening in diabetic patients, and that fatal outcome could be favored by a concurrent metformin excessive plasma concentration. These observations warrant testing for HEV infection in this special population, at least systematically, in the case of an altered general condition and/or liver cytolysis.

## Data Availability

The HEV sequence is available from the NCBI GenBank nucleotide sequence database upon Id. 2613729.
